# Dual inhibition of TGF‐β and PD‐L1: a novel approach to cancer treatment

**DOI:** 10.1002/1878-0261.13146

**Published:** 2022-01-04

**Authors:** James L. Gulley, Jeffrey Schlom, Mary Helen Barcellos‐Hoff, Xiao‐Jing Wang, Joan Seoane, Francois Audhuy, Yan Lan, Isabelle Dussault, Aristidis Moustakas

**Affiliations:** ^1^ Genitourinary Malignancies Branch Center for Cancer Research National Cancer Institute Bethesda MD USA; ^2^ Laboratory of Tumor Immunology and Biology Center for Cancer Research National Cancer Institute Bethesda MD USA; ^3^ Department of Radiation Oncology University of California San Francisco San Francisco CA USA; ^4^ Department of Pathology University of Colorado Aurora CO USA; ^5^ ICREA Vall D’Hebron Institute of Oncology CIBERONC Universitat Autonoma de Barcelona Barcelona Spain; ^6^ EMD Serono Billerica MA USA; ^7^ Department of Medical Biochemistry and Microbiology Science for Life Laboratory Uppsala University Uppsala Sweden; ^8^ Present address: Fusion Pharmaceuticals Boston MA USA

**Keywords:** immune checkpoint inhibitor, PD‐L1, TGF‐β, tumor microenvironment

## Abstract

Transforming growth factor‐β (TGF‐β) and programmed death ligand 1 (PD‐L1) initiate signaling pathways with complementary, nonredundant immunosuppressive functions in the tumor microenvironment (TME). In the TME, dysregulated TGF‐β signaling suppresses antitumor immunity and promotes cancer fibrosis, epithelial‐to‐mesenchymal transition, and angiogenesis. Meanwhile, PD‐L1 expression inactivates cytotoxic T cells and restricts immunosurveillance in the TME. Anti‐PD‐L1 therapies have been approved for the treatment of various cancers, but TGF‐β signaling in the TME is associated with resistance to these therapies. In this review, we discuss the importance of the TGF‐β and PD‐L1 pathways in cancer, as well as clinical strategies using combination therapies that block these pathways separately or approaches with dual‐targeting agents (bispecific and bifunctional immunotherapies) that may block them simultaneously. Currently, the furthest developed dual‐targeting agent is bintrafusp alfa. This drug is a first‐in‐class bifunctional fusion protein that consists of the extracellular domain of the TGF‐βRII receptor (a TGF‐β ‘trap’) fused to a human immunoglobulin G1 (IgG1) monoclonal antibody blocking PD‐L1. Given the immunosuppressive effects of the TGF‐β and PD‐L1 pathways within the TME, colocalized and simultaneous inhibition of these pathways may potentially improve clinical activity and reduce toxicity.

## Graphical abstract

The TGF‐β and PD‐L1 signaling pathways have complementary, nonredundant functions in the tumor microenvironment. Dysregulated TGF‐β signaling suppresses antitumor immunity and promotes cancer fibrosis, epithelial–mesenchymal transition, and angiogenesis, while PD‐L1 restricts immunosurveillance. We review existing strategies for simultaneous inhibition of these pathways, highlighting dual‐targeting agents that may provide colocalized, simultaneous inhibition.

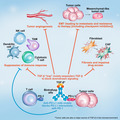

**Abbreviations**
bFGF: basic fibroblast growth factor
CAF: cancer‐associated fibroblast
CTL: cytotoxic T lymphocyte
CTLA‐4: cytotoxic T‐lymphocyte‐associated protein 4
DC: dendritic cell
EGFR: epidermal growth factor receptor
EMT: epithelial–mesenchymal transition
GARP: glycoprotein A repetitions predominant
GM‐CSF: granulocyte–macrophage colony‐stimulating factor
HCC: hepatocellular carcinoma
HMGA2: high mobility group A2
HR: hazard ratio
LAP: latency‐associated peptide
mAb: monoclonal antibody
MDSC: myeloid‐derived suppressor cell
NK: natural killer
NR: not reached
NSCLC: non‐small‐cell lung cancer
ORR: objective response rate
OS: overall survival
PARPi: poly ADP‐ribose polymerase inhibition therapy
PD‐1: programmed death 1
PDGF: platelet‐derived growth factor
PD‐L1: programmed death ligand 1
PR: partial response
RCC: renal cell carcinoma
SCCHN: squamous cell carcinoma of the head and neck
siRNA: small interfering RNA
SOC: standard of care
TAM: tumor‐associated macrophage
TEAE: treatment‐emergent adverse event
TGF‐β: transforming growth factor‐β
TGF‐βR: transforming growth factor‐β receptor
TME: tumor microenvironment
TRAE: treatment‐related adverse event
T_reg_
: regulatory T cell
UC: urothelial carcinoma
VEGF: vascular endothelial growth factor

## 1 The role of TGF‐β in cancer physiology

Transforming growth factor‐β (TGF‐β) is a pleiotropically acting cytokine, of which there are three isoforms that are encoded by different genes that are ubiquitously expressed across tissues. These isoforms activate signaling pathways via type I and type II TGF‐β receptors, and the activity of TGF‐β is regulated at a variety of steps [1, 2]. TGF‐β is secreted by cells in an inactive form attached to a peptide partner (the latency‐associated peptide, LAP). This latent form can undergo isoform‐specific activation through the cleavage of the LAP by extracellular proteases to release active TGF‐β, through integrins (such as ανβ6 or ανβ8) binding to the LAP to exert a physical force to release active TGF‐β, or through modification of the LAP by thrombospondin [3]. The active TGF‐β then binds to the serine/threonine protein kinase receptors TGF‐βRI and TGF‐βRII, sometimes with the aid of the TGF‐βRIII receptor, betaglycan; the activated receptor complex then phosphorylates specific SMADs, a family of signal‐transduction proteins, that form heterotrimeric SMAD complexes. These activated SMAD complexes interact with cell‐specific transcription factors in the nucleus [3]. While SMAD2, SMAD3, and SMAD4 participate in TGF‐β signaling, SMAD6 and SMAD7 inhibit TGF‐β signaling [3]. The resulting signaling pathway can induce a large and diverse set of cellular responses that are highly context‐ and tissue‐specific [3, 4].

Physiologically, TGF‐β maintains immunological self‐tolerance and acts to suppress cancer by regulating epithelial proliferation, apoptosis, and differentiation [3, 5]. TGF‐β signaling undergoes changes during malignant transformation, resulting in TGF‐β functioning as a tumor promoter rather than a suppressor [4, 6]. Aberrant TGF‐β activation and signaling can promote disease progression by stimulating epithelial–mesenchymal transition (EMT), angiogenesis, cancer‐associated fibroblast (CAF) activation, and immunosuppression within the tumor microenvironment (TME) [7, 8, 9]. High expression of TGF‐β in the TME correlates with poor clinical outcome and increased likelihood of metastasis in various tumor types [10, 11].

**Fig. 1:**
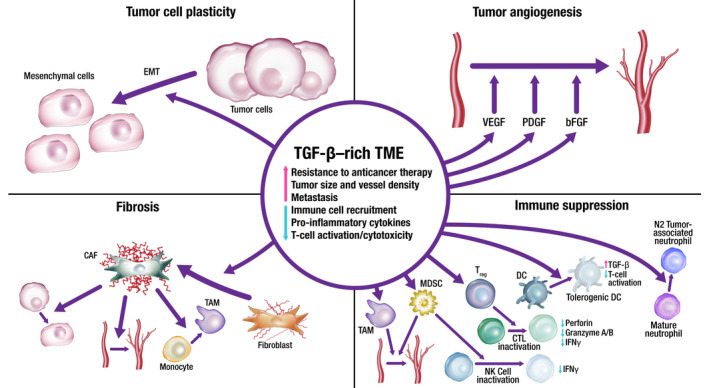
TGF‐β‐rich TME promotes survival mechanisms, including angiogenesis, immune suppression, fibrosis, and tumor cell plasticity. Through these mechanisms, TGF‐β signaling prevents antitumor immune responses, limits drug and immune cell access to the tumor, and promotes resistance to therapy. Through these processes, TGF‐β also promotes invasion and metastasis. bFGF, basic fibroblast growth factor; CAF, cancer‐associated fibroblast; CTL, cytotoxic T lymphocyte; DC, dendritic cell; EMT, epithelial–mesenchymal transition; IFN, interferon; MDSC, myeloid‐derived suppressor cell; NK, natural killer; PDGF, platelet‐derived growth factor; TAM, tumor‐associated macrophage; TGF, transforming growth factor; TME, tumor microenvironment; T_reg_, regulatory T cell; VEGF, vascular endothelial growth factor.

Epithelial–mesenchymal transition is implicated in migration and invasion of cancer cells (Fig. 1) [12]. EMT may increase the number of cancer stem cells [13], and TGF‐β signaling in the TME can further promote this by enabling epithelial cells to acquire stem cell‐like properties [14]. EMT is also associated with resistance to anti‐programmed cell death (ligand) 1 (PD‐[L]1) therapies, targeted therapies, and chemotherapy [15, 16, 17]. Aberrant TGF‐β signaling can upregulate the expression of proangiogenic factors such as vascular endothelial growth factor (VEGF), platelet‐derived growth factor (PDGF), and basic fibroblast growth factor (bFGF), resulting in increased blood vessel density and tumor size [18, 19]. TGF‐β signaling in the TME is associated with the transition of fibroblasts into CAFs that contribute to tumor drug resistance and metastasis [20, 21]. CAFs can remodel the extracellular matrix to influence T‐cell migration and trap T cells in the stroma, as well as express a variety of cytokines that are involved in immune suppression and metastasis: interleukin (IL)‐6, IL‐8, IL‐11, CCL2, PGE2, CXCL12, and TGF‐β [17, 20, 21, 22, 23].

TGF‐β plays an important role in immunity, particularly in immunosuppression, as evidenced by the fact that most immune cells respond strongly to its effects [4]. Immunosuppression by TGF‐β in cancer involves a phenotypic change in a variety of immune cells: dendritic cells, tumor‐associated macrophages (TAMS), tumor‐associated neutrophils, natural killer (NK) cells, myeloid‐derived suppressor cells (MDSCs), regulatory T cells (T_reg_ cells), and cytotoxic T cells (Fig. 1) [8, 9, 23, 24]. TGF‐β is an autocrine survival signal for myeloid precursors and drives their differentiation to highly immunosuppressive MDSCs at the expense of macrophages and dendritic cells [25]. In the TGF‐β‐rich TME, dendritic cells shift into a tolerogenic phenotype, with reduced antigen presentation and ability to activate T cells [26]. Macrophages shift from an inflammatory (M1) to a tumor‐trophic (M2) phenotype to become TAMs. This phenotype expresses proinflammatory cytokines at a reduced rate, while expression of TGF‐β and VEGF are increased [27]. Rather than developing an antitumor phenotype, mature neutrophils in the TME preferentially adopt a phenotype that promotes tumor growth, immunosuppression, metastasis, invasion, and angiogenesis [28]. TGF‐β can suppress the proliferation and cytotoxicity of NK cells and reduce their interferon gamma (IFN‐γ) production. IFN‐γ is an activator of macrophages and stimulates NK cells and neutrophils [3, 24]. Undifferentiated T cells can switch to a T_reg_ phenotype in the presence of TGF‐β; this switch can lead to both the inactivation of effector and cytotoxic T cells and an increase in MDSCs, which subsequently differentiate to tumor‐associated macrophages or tumor‐associated neutrophils [3, 24]. Systemic TGF‐β levels are often increased in people with cancer relative to those in healthy individuals, and elevated TGF‐β levels are associated with poor prognosis and aggressive cancer [27]. However, the use of TGF‐β as a biomarker is complicated by the technological challenges associated with measuring active TGF‐β levels in the TME and the predominance of latent TGF‐β in circulation [29].

## 2 Targeting TGF‐β for cancer therapy

Inhibition of the TGF‐β pathway remains an active area of interest in cancer research, and TGF‐β‐targeted neutralizing antibodies, vaccines, antisense oligonucleotides, and small‐molecule inhibitors have all been investigated in clinical trials of solid tumors [1, 8]. In addition to overcoming immunosuppression, preclinical studies have demonstrated that the blockade of TGF‐β signaling suppresses fibrosis, EMT, and angiogenesis and inhibits tumor growth [7, 8].

The 3 major strategies for targeting TGF‐β are to block expression and activation of TGF‐β, block the binding of TGF‐β to its receptors (including sequestering or ‘trapping’ TGF‐β), or inhibit TGF‐β receptor kinase signaling. Therapies that block TGF‐β activation and expression include gemogenovatucel‐T, a plasmid‐based therapy; SRK‐181, a TGF‐β1 antibody; abituzumab, a pan‐αν integrin antibody; PF‐06940434, an ανβ8 integrin antagonist; and cotsiranib, a small interfering RNA therapeutic that inhibits the expression of TGF‐β1. Gemogenovatucel‐T is a gene therapy consisting of a plasmid of a bifunctional short hairpin RNA that suppresses mature TGF‐β1/2 processing and expresses the immune‐stimulatory cytokine granulocyte–macrophage colony‐stimulating factor [30, 31]. In a phase 1 prospective study, patients receiving gemogenovatucel‐T (*n* = 15) had a 1‐year overall survival (OS) rate of 73% vs 23% in patients not receiving gemogenovatucel‐T (*n* = 13) [32]. No grade ≥ 3 treatment‐related adverse events (TRAEs) were observed in the gemogenovatucel‐T‐treated patients, and the most common TRAEs included grade 1 injection site induration and injection site erythema reported in 12 and 11 patients, respectively. SRK‐181, which inhibits TGF‐β1 activation by targeting regions of the latent TGF‐β complex that mediate cell‐associated activation, may provide a means to selectively block activity in certain cells and is currently being studied in patients with solid tumors (NCT04291079) [33]. Abituzumab blocks the activation of TGF‐β by binding to the αν integrin subunit. In a phase 1/2 trial in patients with metastatic colorectal cancer (*N* = 216), abituzumab therapy in combination with the standard of care (SOC) showed acceptable tolerability, but the primary endpoint of progression‐free survival was not met. However, OS benefit was observed in patients with tumors showing high‐integrin ανβ6 expression in each of the abituzumab dosing arms compared with the SOC; the median OS was not reached (NR) (95% CI, 9.7‐NR months) for the abituzumab 1000 mg + SOC group, 15.0 months (95% CI, 10.5–23.2 months) for the abituzumab 500 mg + SOC group, and 10.2 months (95% CI, 5.8–13.1 months) for the SOC group [34]. High‐integrin ανβ6 expression correlated with worse survival outcomes in the SOC treatment group but correlated with increased survival benefit upon treatment with either abituzumab 1000 mg + SOC (hazard ratio [HR], 0.41 vs 1.58 in the high‐integrin and low‐integrin groups, respectively) or abituzumab 500 mg + SOC (HR, 0.55 vs 1.48 in the high‐integrin and low‐integrin groups, respectively). Treatment‐emergent AEs (TEAEs) occurred in 100% of patients in both the lower‐dose and the higher‐dose abituzumab groups, with the most common TEAEs being diarrhea (65% and 67%, respectively), stomatitis (25% and 30%), and asthenia (21% and 29%). PF‐06940434 is currently under investigation for treatment of patients with advanced or metastatic solid tumors, but data indicating efficacy have yet to be published [35]. Cotsiranib, a TGF‐β1 and COX‐2 small interfering RNA inhibitor, is under investigation in basal cell carcinoma (NCT04669808) and in patients with advanced solid tumors with cholangiocarcinoma, hepatocellular carcinoma, or liver metastases (NCT04676633) [36, 37].

Therapies that block TGF‐β ligand currently in clinical development include neutralizing antibodies (fresolimumab, SAR439459, and NIS793) and a fusion protein that functions as a TGF‐β1/3 ‘trap’ (AVID200) [38, 39, 40, 41]. The pan‐TGF‐β‐neutralizing antibody, fresolimumab, was investigated in patients with advanced melanoma or renal cell carcinoma (*N* = 29); 1 patient experienced a partial response (PR), and six patients experienced stable disease. Gingival bleeding, headache, and epistaxis were the most common TRAEs, with each occurring in 13.8% of patients. Potentially, TGF‐β‐related skin lesions, including actinic keratosis and hyperkeratosis (10.3% each), keratoacanthoma and squamous cell carcinoma of the skin (6.9% each), and basal cell carcinoma (3.4%), were also common [41]. SAR439459 is a pan‐TGF‐β antibody that has demonstrated preclinical activity in human cell lines and murine tumor models, in which treatment inhibited the TGF‐β‐induced suppression of CD8^+^ T cells and the development of T_reg_ cells [42]. In a phase 1b study (NCT02947165), no dose‐limiting toxicities were reported in patients (*N* = 120) with advanced solid tumors receiving the TGF‐β1/2 inhibitor, NIS793 in combination with spartalizumab, and PRs were reported in 4 patients across cohorts. The most common TRAEs were rash (*n* = 15), pruritus (*n* = 10), fatigue (*n* = 9), and nausea (*n* = 8) [43, 44]. AVID200 in syngeneic mouse tumor models has demonstrated activity and increased T‐cell infiltration into the TME [45], as well as antifibrotic activity in a separate model of idiopathic pulmonary fibrosis [46]. In idiopathic pulmonary fibrosis, the accumulation of activated, heterogenous myofibroblasts, analogous to the conversion of fibroblasts to CAFs in cancer, is mediated by TGF‐β signaling [47]. In the phase 1 AVID200‐03 dose‐escalation study (NCT03834662), proinflammatory serum marker levels were increased in a dose‐dependent manner in patients (*N* = 19) receiving AVID200; tumor biopsies showed modulation of TGF‐β signaling and immune activation [48]. Grade 3 TRAEs were reported in 2 patients (diarrhea, lipase elevation, and anemia).

Another class is the oral small‐molecule inhibitors of TGF‐βR kinases, including LY3200882, vactosertib, LY2109761, and galunisertib, that prevent SMAD‐mediated TGF‐β signaling [49, 50, 51, 52]. The TGF‐βRI inhibitor, LY3200882, is currently being investigated in patients with solid tumors (NCT02937272) [49]. Vactosertib, a small‐molecule kinase inhibitor of TGF‐βRI, is being investigated in combination with pembrolizumab in colorectal cancer or gastric/gastroesophageal junction cancer (NCT03724851) [53]; vactosertib is also being investigated in combination with durvalumab in the second‐line treatment of non‐small‐cell lung cancer (NSCLC; NCT03732274) [54]. In a phase 1 dose–escalation study, 35.3% of patients receiving vactosertib at ≥ 140 mg (*n* = 17) achieved stable disease. The most common TRAE was fatigue [52]. In a preclinical study, the TGF‐βRI inhibitor, LY2109761, depleted high CD44 and Id1 glioma‐initiating cells (both indicators of poor prognosis) in human glioblastoma specimens [50]. In a translational study, the TGF‐βRI inhibitor galunisertib was used as a monotherapy to enhance antitumor T‐cell immunity and antigen spreading in a mouse model of breast cancer [51]. Clinically relevant doses of galunisertib were used to enhance the antitumor activity of anti‐PD‐L1 therapy (antimurine PD‐L1 clone), resulting in tumor regression and enhanced T‐cell activation in a murine colorectal cancer model [51].

TGF‐β signaling has been implicated in the development and function of the heart, which may present a challenge for systemic inhibition of TGF‐β as an anticancer therapy [55]. Toxicity concerns have been raised for inhibitors of the TGF‐βRI kinase ALK5, based on preclinical studies that showed increased incidence of heart valve lesions in animals receiving the TGF‐βRI kinase inhibitors AZ12601011 and AZ12799734 [56]. Galunisertib, which belongs to this class, was selected for clinical development because incidence of heart lesions appeared only at very high doses or with continuous treatment for 6 months. Clinical research involving > 300 patients has shown that the animal model toxicities of concern for galunisertib have not been reported in humans when intermittent dosing is applied [57]. An anti‐TGF‐βRII antibody, LY3022859, was under clinical investigation in patients with advanced solid tumors (*N* = 14), but the trial was discontinued due to the absence of clinical efficacy and incidence of cytokine storm, despite prophylactic administration of antihistamines and corticosteroids [58].

## 3 The role of the PD‐L1 pathway in cancer

The PD‐1 receptor and its ligand, PD‐L1, are a critical barrier to antitumor immunity. The PD‐L1 pathway mediates tumor immune evasion by suppressing cell killing by cytotoxic T cells and NK cells that express PD‐1 via expression of PD‐L1 by tumor cells, T_reg_ cells, MDSCs, and macrophages in the TME, resulting in loss of tumor immunosurveillance [59, 60]. Inhibition of the PD‐L1 pathway, by blocking of either the receptor or the ligand, has the potential to disinhibit cytotoxic T cells in the TME, resulting in long‐lasting antitumor activity in subsets of patients across tumor types [59, 60, 61, 62]. However, relieving T cells from inhibition through anti‐PD‐(L)1 therapy initiates a negative feedback loop, stimulating PD‐L1 production by MDSC and subsequent T‐cell reinhibition [60, 62].

Anti‐PD‐(L)1 therapy has changed the treatment landscape for a variety of solid tumor types, including NSCLC, melanoma, squamous cell carcinoma of the head and neck (SCCHN), renal cell carcinoma (RCC), and urothelial carcinoma (UC), due to higher response rates and more manageable toxicity profiles than chemotherapy. Prior to the use of anti‐PD‐(L)1 therapy, second‐line chemotherapy provided response rates of < 10% in NSCLC [63]. In the phase 1 KEYNOTE‐001 study of the PD‐1 inhibitor pembrolizumab in patients with advanced NSCLC (*N* = 495), the objective response rate (ORR) was 19.4%, with a median duration of response of 12.5 months. TRAEs were reported in 70.9% of patients (most commonly fatigue [19.4%], pruritus [10.7%], and decreased appetite [10.5%]). This led to the National Comprehensive Cancer Network recommending pembrolizumab as a second‐line treatment for PD‐L1‐positive NSCLC [64, 65]. Chemotherapy (dacarbazine) was the SOC for metastatic melanoma for 3 decades after its US Food and Drug Administration approval. In the phase 3 CheckMate 066 study in patients with advanced melanoma (*N* = 418), the PD‐1 inhibitor nivolumab had a median OS of 37.5 months in the nivolumab group vs 11.2 months in the dacarbazine group and an ORR of 40.0% (95% CI, 33.3%‐47.0%) vs 13.9% (95% CI, 9.5%‐19.4%) with dacarbazine (odds ratio, 4.06), with 66.7% of nivolumab responders experiencing an ongoing response after 38.4 months [66, 67]. TRAEs were reported in 77.7% and 77.6% in the nivolumab and dacarbazine cohorts, respectively; the most common TRAEs were pruritus (23.8%), diarrhea (18.9%), and rash (18.4%) in the nivolumab cohort. Second‐line chemotherapy provided response rates of 3–13% in patients with recurrent/metastatic SCCHN, but promising results from immune checkpoint inhibitors have led to the approval of pembrolizumab and nivolumab in this setting [68]. In the phase 1b KEYNOTE‐012 study cohort of pembrolizumab in patients with second‐line recurrent/metastatic SCCHN (*N* = 192), the ORR was 18%, and the median duration of response was NR (range, 2+ to 30+ months) [68, 69]. TRAEs occurred in 64% of patients, with the most common TRAEs being fatigue (22%), hypothyroidism (10%), rash (9%), pruritus, and appetite decrease (8% each).

A phase 2 study of nivolumab in patients with metastatic RCC (*N* = 168) showed an ORR of > 20% across all doses, with a large proportion of responders (40%) experiencing an ongoing response at 24 months [70]. Most patients (73%) experienced a TRAE, the most common of which were fatigue (22–35% of patients across dose groups), nausea (10–13% across dose groups), and pruritus (9–11% across dose groups). In a head‐to‐head phase 3 study, the median OS was 25.0 months (95% CI, 21.8‐NR months) for nivolumab vs 19.6 months (95% CI, 17.6–23.1 months) for the mechanistic target of rapamycin inhibitor, everolimus. The ORR was 25% vs 5% (odds ratio, 5.98) [71]. Nivolumab is now recommended by the National Comprehensive Cancer Network for second‐line treatment of RCC [72]. TRAEs occurred in 79% of patients receiving nivolumab, with the most common TRAEs being fatigue (33%), nausea, and pruritus (14% each). Prior to the use of anti‐PD‐(L)1 therapies in UC, the prognosis of patients receiving second‐line chemotherapy was very poor. Pembrolizumab previously showed survival benefit vs chemotherapy in patients with advanced UC in a phase 3 study, with a median OS of 10.3 months (95% CI, 8.0–11.8 months) vs 7.4 months (95% CI, 6.1–8.3 months). TRAEs occurred in 60.9% of patients receiving pembrolizumab; the most common TRAEs were pruritus (19.5%), fatigue (13.9%), and nausea (10.9%) [73]. Although the first‐line SOC for patients with locally advanced or metastatic UC is platinum‐based chemotherapy, it provides limited long‐term benefits because the median progression‐free survival is approximately 6–8 months and OS is approximately 8–15 months, likely due to development of chemoresistance [74, 75, 76]. In the phase 3 JAVELIN Bladder 100 trial, avelumab first‐line maintenance significantly prolonged OS vs best supportive care alone (21.4 months [95% CI, 18.9–26.1 months] vs 14.3 months [95% CI, 12.9–17.9 months]; HR, 0.69) in patients with UC that had not progressed on first‐line chemotherapy. [77] TRAEs were reported in 98.0% of patients receiving avelumab, the most common of which were fatigue (17.7%), pruritus, and urinary tract infection (17.2% each).

## 4 Rationale for the dual inhibition of TGF‐β and PD‐L1

Despite improvements in treatment outcomes across a variety of tumor types, as of 2019, the median ORR for PD‐(L)1 monotherapy in solid tumors is approximately 20%, indicating that a significant unmet need remains [78]. Although durable antitumor responses can be achieved with approved PD‐(L)1 inhibitors, some patients never respond to anti‐PD‐(L)1 therapy (primary refractory), while others develop resistance while receiving anti‐PD‐(L)1 therapy (acquired resistance) [79].

There are three main phenotypes associated with PD‐(L)1 resistance: a TME lacking lymphocytes (‘immune desert’), a TME in which lymphocytes are physically excluded from tumor cells (‘immune‐excluded’), and a TME in which lymphocytes infiltrate the tumor tissue (‘inflamed’) but are inactivated through a negative feedback loop of PD‐L1 signaling, resulting in T‐cell ‘exhaustion’ [80, 81].

TGF‐β and PD‐L1 are nonredundant pathways driving immunosuppression in the TME [9, 60]. TGF‐β can promote PD‐(L)1 resistance by converting conventional T cells to immunosuppressive T_reg_ cells and increasing the survival of myeloid progenitors that differentiate to potent MDSCs [25, 82]. Both of these processes result in increased expression of TGF‐β, while MDSCs express PD‐L1 and drive T_reg_ cell differentiation [25, 82]. In a preclinical study, inhibition of TGF‐β reduced the number of T_reg_ cells, increased the number of effector T cells, and restored sensitivity to anti‐PD‐L1 therapy [83]. In the context of an ‘immune‐excluded’ phenotype, TGF‐β promoted activation of CAFs, which serve as a barrier to T‐cell infiltration of the tumor parenchyma, that has been shown to limit the efficacy of anti‐PD‐L1 therapy in bladder cancer [17]. Lastly, T‐cell ‘exhaustion’ is a phenomenon in which tumors exhibit robust immune infiltration within the TME (as in the ‘inflamed’ phenotype) but are ineffective in controlling tumor growth. PD‐L1 binding of the receptor PD‐1 on immune cells can promote a negative feedback loop whereby T cells express other checkpoint molecules such as TIGIT, LAG‐3, and TIM3 to limit cytotoxic activity [80].

**Fig. 2:**
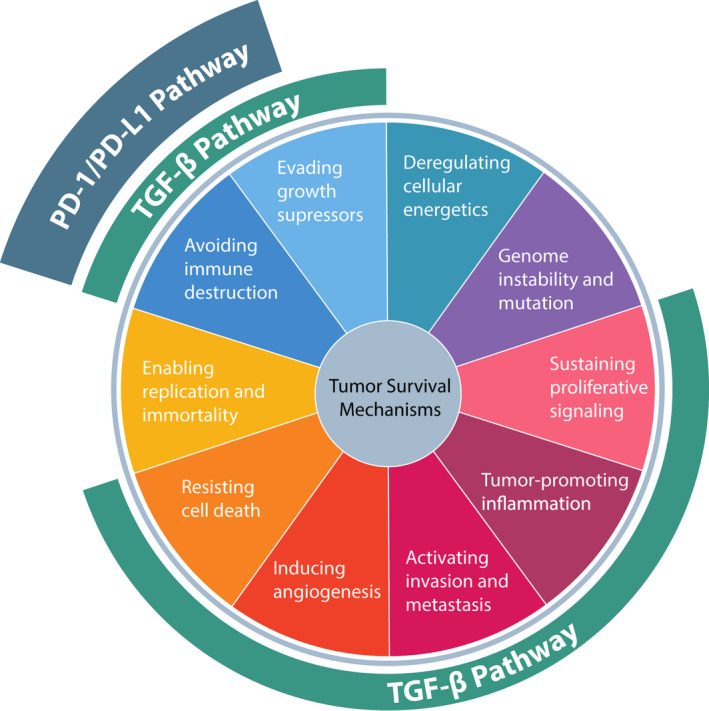
TGF‐β and PD‐L1 signaling pathways are implicated in overlapping but nonredundant tumor survival mechanisms, such that simultaneous inhibition may enhance antitumor activity over inhibition of either pathway alone. PD‐1, programmed death 1; PD‐L1, programmed death ligand 1; TGF, transforming growth factor.

Given the importance of the TGF‐β and PD‐L1 pathways in the development of cancer, the simultaneous inhibition of these pathways may potentially enhance the antitumor activity observed when each pathway is targeted alone (Fig. 2). A recent analysis of plasma levels of soluble TGF‐β and PD‐L1 in 90 patients treated with first‐line chemotherapy found that there was a positive correlation between TGF‐β and PD‐L1 at baseline and following treatment and that an increase in soluble TGF‐β levels following chemotherapy was associated with worse prognosis [84]. In addition, in a biomarker analysis of pretreatment tumor samples from the phase 2 IMvigor210 trial, high expression of TGF‐β was associated with lack of response to PD‐L1 blockade with atezolizumab [17]. Inhibition of TGF‐β may thus remove a barrier in solid tumors to immune checkpoint inhibitor‐based therapy [85]. Furthermore, in PD‐L1‐positive tumors, the TGF‐β is concentrated in the TME; hence, a single molecule targeting PD‐L1 and TGF‐β may ensure suppression of TGF‐β signaling in the TME compared with independent dual combination therapies. A preclinical study evaluated the anticancer efficacy of 2 bifunctional molecules: 1 targeting TGF‐β and PD‐L1 and 1 targeting TGF‐β and CTLA‐4. Both bifunctional molecules were superior to their parent immune checkpoint inhibitors (atezolizumab and ipilimumab, respectively) used alone or in combination with the TGF‐βRII domain in causing tumor regression in murine models [86]. An additional bispecific antibody for TGF‐β and PD‐L1, YM101 was recently described in a preclinical study. YM101 demonstrated the ability to bind all 3 isoforms of TGF‐β, and its antitumor activity was better than the combination of anti‐TGF‐β and anti‐PD‐L1 treatments in mouse tumor models [87].

**Fig. 3:**
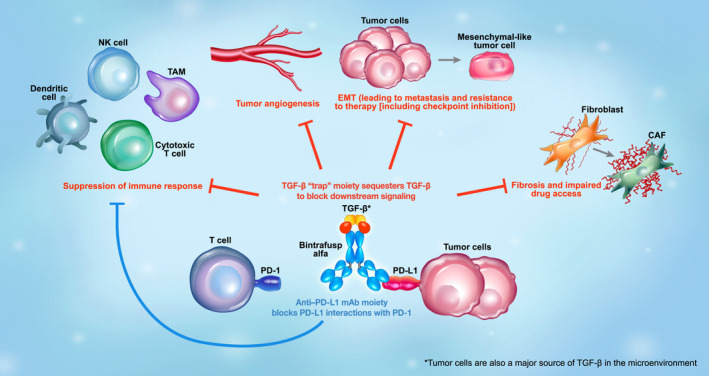
Mechanism of action of bintrafusp alfa, a first‐in‐class bifunctional fusion protein composed of the extracellular domain of TGF‐βRII to function as a TGF‐β ‘trap’ fused to a human IgG1 antibody blocking PD‐L1. Through colocalized, simultaneous inhibition of these pathways, bintrafusp alfa has the potential to enhance immune cell access to the tumor, limit metastasis, and improve response to anticancer therapy. Bintrafusp alfa has the potential to inhibit angiogenesis through suppression of TGF‐β activity via stromal modulation and may restore normal vascular homeostasis, thereby enhancing drug delivery and T‐cell infiltration into the TME. CAF, cancer‐associated fibroblast; EMT, epithelial–mesenchymal transition; NK, natural killer; PD‐1, programmed death 1; PD‐L1, programmed death ligand 1; TAM, tumor‐associated macrophage; TGF, transforming growth factor; TME, tumor microenvironment.

Preclinical studies of bintrafusp alfa, a first‐in‐class bifunctional fusion protein composed of the extracellular domain of the TGF‐βRII receptor to function as a TGF‐β ‘trap’ fused to a human immunoglobulin G1 monoclonal antibody blocking PD‐L1 (Fig. 3), also demonstrated that simultaneous inhibition of both pathways using a bifunctional approach resulted in superior antitumor activity compared with either the TGF‐β ‘trap’ or anti‐PD‐L1 antibody [88]. In the absence of a specific anti‐PD‐L1 moiety, the TGF‐β trap reduced the plasma levels of TGF‐β1 but did not decrease the TGF‐β‐dependent signaling in the TME [89], indicating the importance of the bifunctional nature of bintrafusp alfa to ensure that suppression of TGF‐β occurs only in the TME. In preclinical studies, bintrafusp alfa sequestered all 3 isoforms of TGF‐β in the TME and bound efficiently and specifically to PD‐L1 (both *in vitro* and *in vivo*). This resulted in superior tumor regression compared with a TGF‐β ‘trap’, an anti‐PD‐L1 antibody, or the combination of both [88]. The fact that the isolated TGF‐βRII ectodomain shows high binding affinity toward TGF‐β1 and TGF‐β3 but not TGF‐β2, and yet bintrafusp alfa neutralizes all three circulating isoforms in both mice and humans [88, 90], suggests an avidity effect. This is because both of the TGF‐βRII ectodomain moieties, configured in an obligatory dimeric structure in bintrafusp alfa, can simultaneously bind to each protomer of the TGF‐β homodimer, providing avidity. A radiolabeling study of bintrafusp alfa showed that while the spleen and lung can attract PD‐L1‐targeting antibodies, thereby limiting biodistribution, bintrafusp alfa localizes to the TME *in vivo* [91]. Preclinical data have shown reduced cancer fibrosis with bintrafusp alfa, which can result in (a) enhanced immune cell access to the tumor, (b) restored drug access to the tumor, and (c) reduced metastatic potential of the tumor [88, 89, 92]. Bintrafusp alfa has the potential to inhibit angiogenesis through suppression of TGF‐β activity via stromal modulation and may restore normal vascular homeostasis, thereby enhancing drug delivery and T‐cell infiltration into the TME [93, 94, 95]. Likewise, bintrafusp alfa may reduce the expression of VEGF and subsequent angiogenesis by sequestering TGF‐β [83].

Given the effect of radiation therapy to increase TGF‐β activation and immunogenicity by antigen release, bintrafusp alfa may be a suitable combination partner for radiation therapy by counteracting TGF‐β signaling, increasing infiltration of CD8^+^ T cells, and enhancing the abscopal effect [88, 96, 97, 98]. In addition, bintrafusp alfa may reduce radiation‐induced fibrosis, which has been linked to treatment resistance [88, 99]. Similarly, preclinical data support synergistic effects with the combination of bintrafusp alfa and chemotherapy because TGF‐β inhibition may normalize the extracellular matrix and improve drug delivery and resistance through effects on fibrosis, EMT, and angiogenesis and elimination of chemotherapy‐resistant cancer stem‐like cells [88, 100]. Simultaneous targeting of 2 nonredundant immunosuppressive pathways may result in superior antitumor activity.

## 5 Treatment strategies for the dual inhibition of TGF‐β and PD‐L1

### 5.1 Approaches combining anti‐TGF‐β and anti‐PD‐(L)1 agents

Many TGF‐β‐targeting therapies are under clinical investigation in combination with anti‐PD‐(L)1 therapies, especially in tumor types that have had poor responses to anti‐PD‐(L)1 monotherapies. For advanced gynecological cancers, gemogenovatucel‐T is under investigation alone or in combination with atezolizumab (NCT03073525) and in combination with durvalumab (NCT02725489) [30, 101]. Galunisertib in combination with durvalumab was studied in patients with metastatic pancreatic cancer (NCT02734160) [102]. An initial analysis reported 1 PR among 42 evaluable patients [103]. Seven grade ≥ 3 TRAEs occurred; increased aspartate aminotransferase and γ‐glutamyltransferase were the most common events. Galunisertib in combination with nivolumab is being investigated in patients with advanced refractory solid tumors, NSCLC, and hepatocellular carcinoma (NCT02423343) [104]. In the hepatocellular carcinoma cohort, two PRs were reported among 47 patients [105]. The most common TEAEs were fatigue (33.6%), anemia (25.5%), peripheral edema (22.8%), and abdominal pain (21.5%); grade 3/4 TRAEs were much less frequent, with neutropenia (2.7%), fatigue, anemia, increased bilirubin, hypoalbuminemia, and embolism (1.3% each) being the most common events. LY3200882, either alone or in combination with chemotherapy, radiation, or anti‐PD‐L1 therapy (NCT02937272), is currently under investigation in a phase 1 study of patients with solid tumors [49]; 1 PR was reported among 30 patients [106]. No grade ≥ 3 TRAEs were reported; the most common TRAEs were thrombocytopenia, acneiform dermatitis, rash, and constipation (two patients each). SAR439459 is currently under investigation in a phase 1b study (NCT03192345), either alone or in combination with cemiplimab, an anti‐PD‐1 agent, for treatment of patients with advanced solid tumors [38]. NIS793 is under investigation in a phase 1 study (NCT02947165), either alone or in combination with spartalizumab, an anti‐PD‐L1 agent, for treatment of patients with advanced solid tumors [43]. In a phase 1 study (NCT04152018), PF‐06940434 is currently under investigation as a monotherapy and as a combination partner with an anti‐PD‐L1 therapy (PF‐06801591) for treatment of patients with advanced or metastatic solid tumors [35]. Together, these results provide evidence that dual inhibition of TGF‐β and PD‐(L)1 may provide a more manageable safety profile in line with PD‐(L)1 monotherapy than that observed for TGF‐β monotherapy. However, the limited efficacy observed with these combinations suggests that further improvements may be possible through dual‐targeting agents that localize TGF‐β inhibition within the TME. Additionally, no biomarkers of response to TGF‐β inhibition have been identified to date. Based on preclinical studies that identified distinct cellular and molecular profiles of responders vs nonresponders to dual inhibition of TGF‐β and PD‐L1 [107, 108], further investigation is warranted into immune phenotype and TGF‐β‐related gene expression signatures, as well as genomic biomarkers.

### 5.2 Dual‐targeting agents

To date, there are 2 single‐molecule TGF‐β/PD‐L1 inhibitors in clinical development: bintrafusp alfa and SHR‐1701, another TGF‐βRII/PD‐L1 bifunctional fusion protein. SHR‐1701 is currently being evaluated in phase 1/2 studies in patients with locally advanced/metastatic solid tumors (Table 1) [109, 110, 111, 112, 113]. To our knowledge, bintrafusp alfa is the most developed single‐molecule therapy, with multiple phase 2 trials in biliary tract cancer, NSCLC, cervical cancer, and other solid tumors (Table 1) [88, 89, 90, 98, 114, 115, 116, 117, 118, 119, 120].

**Table 1:** Overview of therapies currently under investigation targeting TGF‐β and PD‐L1. CTLA‐4, cytotoxic T‐lymphocyte‐associated protein 4; EGFR, epidermal growth factor receptor; GARP, glycoprotein A repetitions predominant; GM‐CSF, granulocyte–macrophage colony‐stimulating factor; HCC, hepatocellular carcinoma; HMGA2, high mobility group A2; mAb, monoclonal antibody; NSCLC, non‐small‐cell lung cancer; PARPi, poly ADP‐ribose polymerase inhibition therapy; PD‐1, programmed death 1; PD‐L1, programmed death ligand 1; siRNA, small interfering RNA; TGF‐β, transforming growth factor‐β; TGF‐βR, transforming growth factor‐β receptor; VEGF, vascular endothelial growth factor.

TGF‐β inhibitor	Combination partner(s)	Mechanism of action	Clinical trial ID	Phase	Patient population	Primary completion date
Bintrafusp alfa	None	Bifunctional TGF‐β ‘trap’/anti‐PD‐L1 mAb	NCT03631706 [130]	3	Advanced NSCLC with high PD‐L1 tumor expression	Trial discontinued January 19, 2021 [131]
Bintrafusp alfa	Chemotherapy	Bifunctional TGF‐β ‘trap’/anti‐PD‐L1 mAb	NCT04066491 [114]	2	Locally advanced/metastatic biliary tract cancer	Trial discontinued Aug 23, 2021 [132]
Bintrafusp alfa	None	Bifunctional TGF‐β ‘trap’/anti‐PD‐L1 mAb	NCT03833661 [133]	2	Platinum‐experienced, locally advanced/metastatic biliary tract cancer	November 2020
Bintrafusp alfa	None	Bifunctional TGF‐β ‘trap’/anti‐PD‐L1 mAb	NCT04489940 [134]	2	HMGA2‐expressing triple negative breast cancer	February 2023
Bintrafusp alfa	None	Bifunctional TGF‐β ‘trap’/anti‐PD‐L1 mAb	NCT04246489 [135]	2	Advanced unresectable or metastatic cervical cancer	April 2022
Bintrafusp alfa	Concurrent chemoradiation	Bifunctional TGF‐β ‘trap’/anti‐PD‐L1 mAb	NCT03840902 [116]	2	Unresectable stage III NSCLC	May 2023
Bintrafusp alfa	Chemotherapy	Bifunctional TGF‐β ‘trap’/anti‐PD‐L1 mAb	NCT03840915 [115]	1b/2	Stage IV NSCLC	January 2022
Bintrafusp alfa	None	Bifunctional TGF‐β ‘trap’/anti‐PD‐L1 mAb	NCT02517398 [117]	1	Advanced solid tumors	September 2022
Bintrafusp alfa	None	Bifunctional TGF‐β ‘trap’/anti‐PD‐L1 mAb	NCT02699515 [118]	1	Advanced solid tumors	September 2022
Bintrafusp alfa	Chemotherapy and radiation or bevacizumab (anti‐VEGF)	Bifunctional TGF‐β ‘trap’/anti‐PD‐L1 mAb	NCT04551950 [136]	1	Locally advanced or advanced cervical cancer	May 2022
Bintrafusp alfa	None	Bifunctional TGF‐β ‘trap’/anti‐PD‐L1 mAb	NCT04349280 [137]	1b	Platinum‐experienced metastatic or locally advanced/unresectable urothelial cancer	September 2022
Galunisertib	Nivolumab (anti‐PD‐1)	TGF‐βRI inhibitor	NCT02423343 [104]	1b/2	Advanced refractory solid tumors, recurrent/refractory NSCLC, HCC	December 2018
Galunisertib	Durvalumab (anti‐PD‐L1)	TGF‐βRI inhibitor	NCT02734160 [102]	1b	Metastatic pancreatic cancer	August 2018
LY3200882	Chemotherapy, radiation, and/or LY3300054 (anti‐PD‐L1)	TGF‐βRI inhibitor	NCT02937272 [49]	1	Solid tumors	February 2020
NIS793	Spartalizumab (anti‐PD‐1)	Anti‐TGF‐β mAb	NCT02947165 [43]	1	Advanced malignancies	June 2021
NIS793	Chemotherapy +/‐ spartalizumab	Anti‐TGF‐β mAb	NCT04390763 [40]	2	Metastatic pancreatic ductal adenocarcinoma	January 2023
PF‐06940434	PF‐06801591 (anti‐PD‐1)	α‐ν/β‐8 integrin inhibitor	NCT04152018 [35]	1	Advanced/metastatic solid tumors	January 2024
SAR439459	Cemiplimab (anti‐PD‐1)	Pan‐TGF‐β inhibitor	NCT03192345 [38]	1b	Advanced solid tumors	April 2023
SHR‐1701	None	TGF‐β‐RII/PD‐L1	NCT03710265 [109]	1	Locally advanced/metastatic solid tumors	November 2019
SHR‐1701	None	TGF‐β‐RII/PD‐L1	NCT04324814 [113]	1	Advanced solid tumors	May 2022
SHR‐1701	Radiotherapy	TGF‐β‐RII/PD‐L1	NCT04560244 [112]	2	Metastatic NSCLC	September 2022
SHR‐1701	None	TGF‐β‐RII/PD‐L1	NCT03774979 [111]	1	Advanced solid tumors	July 2021
SHR‐1701	None	TGF‐β‐RII/PD‐L1	NCT04282070 [110]	1b	Recurrent/metastatic nasopharyngeal carcinoma	April 2022
Gemogenovatucel‐T	Durvalumab (anti‐PD‐L1)	TGF‐β1/2 inhibitor, GM‐CSF expresser	NCT02725489 [101]	2	Advanced breast and gynecological cancers	December 2019
Gemogenovatucel‐T	Atezolizumab (anti‐PD‐L1)	TGF‐β1/2 inhibitor, GM‐CSF expresser	NCT03073525 [30]	2	Advanced gynecological cancers	January 2021
Vactosertib	Durvalumab (anti‐PD‐L1)	TGF‐βRI inhibitor	NCT03732274 [54]	1b/2	Advanced NSCLC	October 2022
Vactosertib	Pembrolizumab (anti‐PD‐1)	TGF‐βRI inhibitor	NCT03724851 [53]	1b/2	Metastatic colorectal or gastric cancer	June 2021
AVID200	None	TGF‐β1/3 ‘trap’	NCT03834662 [39]	1	Advanced or metastatic solid tumors	February 2020
Cotsiranib	None	TGF‐β1/COX‐2 siRNA inhibitor	NCT04676633 [36]	1	Advanced solid tumors with cholangiocarcinoma, HCC, or liver metastases	March 2024
Cotsiranib	None	TGF‐β1/COX‐2 siRNA inhibitor	NCT04669808 [37]	2	Basal cell carcinoma	December 2021
SRK‐181	Anti‐PD‐(L)1 antibody therapy	Anti‐TGF‐β mAb	NCT04291079 [138]	1	Advanced or metastatic solid tumors	December 2021

In a phase 1 dose–escalation and dose–expansion study (NCT03710265), no dose‐limiting toxicities were observed, and the maximum tolerated dose was not reached for patients (*N* = 49) with advanced solid tumors receiving SHR‐1701; in patients who were evaluable for efficacy (*n* = 45), the ORR was 17.8% (95% CI, 8.0%–32.1%), with 7 of 8 responses ongoing. The most common TRAEs (incidence > 15%) were increased alanine aminotransferase/aspartate aminotransferase, anemia, hypothyroidism, and increased bilirubin/conjugated bilirubin [121]. In an expansion cohort of a phase 1b study (NCT03774979), patients with epidermal growth factor‐positive NSCLC (*N* = 27; 24 were evaluable for efficacy) receiving SHR‐1701 had an ORR of 16.7% (95% CI, 4.7%‐37.4%). Grade 3 TRAEs occurred in 7.4% of patients, including anemia, hypokalemia, and asthenia (3.7% each) [122].

Results from 2 phase 1 studies (NCT02517398 and NCT02699515) of > 670 patients treated with bintrafusp alfa demonstrated promising clinical activity in various expansion cohorts of advanced solid tumors [90, 120, 123, 124, 125, 126, 127, 128, 129]. Durable responses of > 6 months were reported in multiple advanced solid tumor types, and response rates were compared favorably with historical data for anti‐PD‐(L)1 therapies in the same tumor type, although no head‐to‐head studies have been conducted. Additionally, the safety profile appeared manageable and consistent with colocalized, simultaneous inhibition of the TGF‐β and PD‐L1 pathways. The most common TRAE preferred terms were rash maculopapular, pruritus, rash, asthenia, and hypothyroidism. TRAEs leading to discontinuation of bintrafusp alfa occurred at a rate of 6–20%. Skin lesions, including those that have been observed with fresolimumab, were observed at a rate of 3–13.3% across eight expansion cohorts.

Interestingly, the cardiac toxicity observed in animals treated with ALK5 inhibitors (AZ12601011 and AZ12799734) [56] and cytokine release observed with anti‐TGF‐βRII antibody (LY3022859) in patients with advanced solid tumors [58] have not been seen in preclinical or clinical trials with bintrafusp alfa. Simultaneous, colocalized inhibition of TGF‐β and PD‐L1 may explain why these TRAEs are not observed in patients treated with bintrafusp alfa, as localization of TGF‐β inhibition to the TME may prevent AEs associated with systemic inhibition of TGF‐β.

Finally, while dual‐targeting agents have demonstrated significant advantages over combinations of TGF‐β and PD‐L1 inhibitors in the clinical setting [81, 82], larger head‐to‐head clinical trials are necessary to confirm these findings. Based on an increasing number of reports that implicate the actions of other TGF‐β family ligands (e.g., activins) in malignancies, future development is also warranted for alternative bifunctional molecules with specificity for TGF‐β family ligands in combination with immune checkpoint inhibitors. Additionally, investigation into sequential inhibition of TGF‐β and PD‐(L)1 (or PD‐[L]1 and TGF‐β) would help to explain the complex biologic interplay of these two pathways.

## 6 Conclusions

Tumor‐promoting activities of TGF‐β within the TME, which include EMT, fibrosis, angiogenesis, and immunosuppression, are nonredundant with the tumor‐evasive mechanisms mediated by PD‐L1; hence, simultaneous inhibition of TGF‐β and PD‐L1 pathways may allow for increased overall efficacy compared with independent blockade of either pathway alone. A single‐molecule therapy targeting both pathways has the potential for a localized optimal suppression of TGF‐β within the TME, a benefit that is not provided by combining independent therapies, as evidenced by the superior preclinical activity of bintrafusp alfa compared with dual inhibition by two molecules. Based on its proposed mechanism of action, the bifunctional nature of bintrafusp alfa and other dual‐targeting agents provides colocalized, simultaneous inhibition of the TGF‐β and PD‐L1 pathways within the TME and represents an attractive potential therapy for patients with advanced solid tumors.

## Conflict of interest

JLG reports a collaborative research and development agreement between the National Cancer Institute (NCI) and EMD Serono, Billerica, MA, USA, a patent with the NCI entitled ‘Combination PDL1 and TGF‐beta blockade in patients with HPV+ malignancies’, unpaid membership on the Data Safety Monitoring Board for bintrafusp alfa, and an unpaid position of NCI Liaison to the Board of the Society for Immunotherapy of Cancer. J Schlom reports a collaborative research and development agreement between the NCI and EMD Serono, Billerica, MA, USA. MHBH reports grants from Roche‐Genentech, Innovation Pathways, NCI, and Varian Medical Systems; consulting fees from Telos Inc. and Innovation Pathways; honoraria from the Society for Immunotherapy in Cancer; conference chairmanship for American Association for Cancer Research; advisory committee memberships for Genentech and EMD Serono, Billerica, MA, USA; a patent entitled ‘DNA Damage Repair Deficit in Cancer Cells’; and receipt of research drugs from Innovation Pathways. FA and YL report employment at EMD Serono, Billerica, MA, USA. ID reports previous employment at EMD Serono, Billerica, MA, USA. XJW, J Seoane, and AM do not have any conflicts of interest to disclose.

## Author contributions

JLG, J Schlom, XJW, AM, MHBH, J Seoane, FA, YL, and ID conceptualized the study. JLG, J Schlom, XJW, AM, MHBH, J Seoane, FA, YL, and ID wrote the original draft. JLG, J Schlom, XJW, AM, MHBH, J Seoane, FA, YL, and ID wrote, reviewed, and edited the manuscript.

## Data Availability

Any requests for data by qualified scientific and medical researchers for legitimate research purposes will be subject to the Data Sharing Policy of the healthcare business of Merck KGaA, Darmstadt, Germany. All requests should be submitted in writing to the data sharing portal of the healthcare business of Merck KGaA, Darmstadt, Germany (https://www.merckgroup.com/en/research/our‐approach‐to‐research‐and‐development/healthcare/clinical‐trials/commitment‐responsible‐data‐sharing.html). When the healthcare business of Merck KGaA, Darmstadt, Germany, has a coresearch, codevelopment, or comarketing or copromotion agreement, or when the product has been out‐licensed, the responsibility for disclosure might be dependent on the agreement between parties. Under these circumstances, the healthcare business of Merck KGaA, Darmstadt, Germany, will endeavor to gain agreement to share data in response to requests.
